# Book Reviews

**Published:** 1809-12

**Authors:** 


### CRITICAL ANALYSIS

OF THE

### RECENT PUBLICATIONS

ON THE

### DIFFERENT BRANCHES OF PHYSIC, SURGERY, AND MEDICAL PHILOSOPHY.

---

*Observations on some of the most frequent and important Diseases of the Heart; on Aneurism of the Thoracic Aorta; on preternatural Pulsation in the Epigastric Region; and on the unusual Origin and Distribution of some of the large Arteries of the Human Body. Illustrated by Cases.* By ALLAN BURNS, Member of the Royal College of Surgeons, London; and Lecturer on Anatomy and Surgery, Glasgow.

THIS work appears to have made part of the author's lectures, when engaged in demonstrating the important organ to which it refers. As part of such a series, it may be said to contain much information where originality is not expected. We shall, however, avail ourselves of this opportunity of bringing before our readers a subject, which several late communications in this and other Journals have rendered particularly interesting.

The first section is a kind of introduction to the subject, in which the diseases of the heart are divided into three classes.

"The first class, containing the sympathetic affections of the heart, or those diseases which are dependent on the consent which this organ has with other parts of the body;

"The second, those affections which are brought on by such malconformations of the heart, as give rise to a mixture of the venous with the arterial blood; and,

"The third, those diseases induced by some organic affection of the heart which mechanically impairs the circulation, but does not necessarily alter the composition or properties of the blood.

"It is not my intention to lay before you any remarks at this time on the first of these classes; and in treating of the other two, I shall endeavour to select only the most frequent and important diseases for particular discussion, and chiefly such as I have myself had an opportunity of seeing.

"The symptoms characterizing the second class of diseases of the heart, may be produced not only by malconformation of this order, but also to a certain degree, by whatever offers a mechanical obstruction to the performance of the respiratory function. Thus, extensive and close adhesion of the pleura pulmonalis to the pleura costalis, will, as we learn from an interesting case by Dr. Marcet, induce symptoms which mark a deficiency of the arterial principle in the blood. Whatever, also, has a tendency to encroach on the bronchial cells, will, according to the extent of the comcompression, produce symptoms which indicate, if I may be permitted to use the uncouth term, imperfect arterialization of the blood. Hence the bloated livid countenance in pulmonic anasarca, and the inflated purple face so universally attendant on pneumonia or inflammation of the lungs. In these diseases, obscure symptoms are produced of an affection of the heart, without any real disease of this organ. The same takes place when the lungs are compressed by effusion of water or secretion of pus into the chest, by solid tumors formed in the thorax, by enlargement of the abdominal viscera, or by accumulation of water in the belly. These are only temporary, and indirect causes, producing a morbid state of the circulating fluids; but the same diseased condition of the blood, is permanently kept up by several malconformations of the heart.

"From the enumeration of the causes producing a deficiency of the arterial principle in the blood, it will, at first sight, appear to you, that they are divisible into two classes. In the first class, this state of the blood is occasioned by the existence of some mechanical obstacle to the action of the lungs, by which the due arterialization of the blood is prevented. But, in the second, which more immediately interests us, the same state of the circulating fluids is induced by a direct mixture of the venous with the arterial blood. You will therefore readily understand, that in the one case, the blood is duly sent into the lungs, to be rendered arterial, but owing to the deficiency of oxygen, this process is imperfectly performed; in the other, owing to some malconformation of the heart, or large vessels, a part of the blood passes directly from the venous into the arterial circulation. Although, therefore, the proper quantity of air may in the latter case enter the bronchiæ, still its presence in them is rendered of no avail by the peculiar formation of the vascular system.

"The symptoms denoting a mixture of the venous with the arterial blood, do not, except where the preponderance of the former is very great, make their appearance for some weeks, or often months, after birth; but, as soon as they occur, they indicate clearly the nature of the affection. The breathing is performed with difficulty, the parts covered by the delicate epidermis lose their natural colour, and assume a livid tint, while the limbs and trunk become of a leaden hue. The child at the same time is averse to motion, which aggravates the symptoms, is affected with *a short suffocating or stifling cough, like that in peripneumony or hooping cough.* The expectoration is but slight, although occasionally blood is brought up by the violence of the exertion. The animal functions are all of them languid, the bowels are torpid, the nervous system is depressed, and the heat of the body is reduced below what it naturally is; such are the permanent effects produced by imperfectly arterialized blood. The symptoms do not however remain constantly in this state, but are subject, after irregular intervals, to exacerbations, during which the heart palpitates, tates, the chest feels constricted, the breathing is oppressed, the head is pained, the eyes are prominent, the lips, ears, nose, tongue, and the parts beneath the nails, become very dark in their colour; nay, in cases of extreme urgency, they acquire a purplish black hue. The anxiety is insufferable, the urine and fæces come away involuntarily, the body feels cold to a by-stander, and appears as if it were on the eve of parting with life. The arterial system acts feebly, the jugular veins are turgid, the countenance is inflated, and resembles that of a person suffering strangulation; the convulsive motions of the limbs indicate the greatest agitation, both of body and mind. This state cannot long remain in full force; therefore, after a shorter or longer continuance, the system falls into a state of quiescence, from which it gradually emerges, continues languid and torpid, and the surface is permanently of a darker colour than it ought to be. On the application of any trivial, and frequently without any obvious cause, the train of symptoms already mentioned recur. In proportion as the child advances in years, the paroxysms become more frequent, more violent, and the remissions less perfect, till at last, upon the supervention of dropsical symptoms, the constitution is completely worn out."

We are obliged to stop here, in order to caution our readers against the danger that attends this mode of compiling, in which the author thinks it enough to transcribe what others have transcribed before him, and what his successors think it sufficient to transcribe again. Where can be the similitude between a *short* stifling cough and the hooping cough? Or in what, except the shortness of the first, do either of them resemble the cough in peripneumonia?

The occasional paroxysm of distressing dyspnœa is extremely well described; but there is a most important omission in the account of the sequel. After each one of these periods of apparent strangulation, it should have been remarked, that for a time the patient loses his purple colour, and becomes of a more bright vermillion than is consistent with sound health; very much resembling the delicate blush which attends a paroxysm of hectic fever. This change might be expected, from the well-marked actions which are taking place throughout the whole fit: it is not, however, from such a surmise, but from actual observation, that we mention it. The description of the progress, and even of the cessation, of the paroxysm, is in all other respects unexceptionable.

" It is worthy of observation, that in the diseases belonging to this class, when the patient is apparently just expiring, he is in reality bettering his condition. Mr. John Bell explains with accuracy and spirit, the changes which take place during the paroxysm. The common respiration, as he observes, will not suffice; the venous blood is gaining on the arterial system; therefore, the lungs must be as completely emptied as possible; the air is accordingly forcibly expelled, the child sinks exhausted, soon he breathes again, but expires with equal exertion as formerly. This struggle continues for a length of time; at last, respiration seems suspended; during this suspension, the surface gradually loses its livid colour, and becomes more of the pale leaden hue. Then the patient fetches a deep sigh, recovery begins, and goes on apace, till at length he is as well as circumstances will permit. While these phenomena are going on, we learn from Mr. Bell, that the child is expelling portion after portion of the contaminated air from the lungs, till finally he succeeds in introducing such a quantity of pure air, as will in some measure restore the balance of arterial and venous blood. Nor is it only by calling into forcible action the natural auxiliary muscles of expiration, that the patient endeavours to make way for the introduction of an extra quantity of atmospheric air into the lungs; he also lessens the capacity of the chest, if he be very young, by turning on his belly, or if he be older, he presses his breast firmly against a table, or any solid object. By these means, which an ignorant spectator considers as of no value, the contaminated air is driven out, new air admitted, relief obtained, and on every occurrence of the paroxysm, the patient has recourse to similar means."

In further considering the effect of such malformations of the heart, as tend to admit venous blood into the circulation without first passing the lungs, the following division is made:

" Species first.—Where the aorta originates equally from the right and left ventricle.

" Species second.—Where the foramen ovale and ductus arteriosus remain open.

" Species third.—Where the ductus arteriosus is obliterated, but the foramen ovale remains pervious; or a preternatural opening leads from the one ventricle to the other.

" Species fourth.—Where the pulmonary artery is impervious at its origin, but still receives a small portion of blood by a retrograde action of the ductus arteriosus.

" Species fifth.—Where the heart merely consists of two cavities, an auricle and ventricle, and where from the latter, a vessel originates which afterwards divides into two branches, one to the lungs, the other to the body.

" Species sixth.—Where the mitral valve is malformed, leaving merely a small opening, leading from the left auricle into the ventricle."

Each of these is illustrated by cases from different writers, and by such as have occurred to the author.

In the section, " on the uniform enlargement and dilatation of the heart," the author begins, as if he intended to mark all the various shades which distinguish the enlarged from the merely dilated heart. We meet only with a few general remarks, in which the attempt is made of ascertaining the disease by the symptoms during life; by the pulse, breathing, and general state of the health. For our own parts, we confess we have not yet been fortunate enough to ascertain these distinctions; yet we have neither to accuse ourselves of want of industry or opportunity. The enormous increase of the muscular part of the heart, without any previous difficulty in the passage of the blood through the arteries, is, we believe, a rare case. In some cases of ossification of the valves, we have seen the heart, both in its cavities and muscular fibres, considerably enlarged; in others, the whole organ has been much diminished, and apparently enfeebled. Were we inclined to theorize with boldness, we should easily explain both these appearances. In the first, we should suppose that the increased difficulty in the first branches of circulation, imposing a greater exertion on the heart, its fibres had increased, as the muscular fibres of the bladder are increased under difficulties of passing the urine. In the second case we may believe, and with some well-founded histories to support us, that the great pain attending every the slightest exertion, had induced the patient, perhaps, for a succession of years, to avoid every means by which the circulation would be increased; and, consequently, that the heart having been kept as quiescent as possible, its fibres had become thinner and weaker, like other muscles when less used. We mention these only as general remarks; but in a work professedly written on diseases of the heart, we expected to have seen something concerning that connection between acute rheumatism and the dilated heart, of which so many instances have lately been recorded.

Some observations follow, on the partial dilatation of the heart; that is, a dilatation affecting one side, or a single cavity. This is illustrated by a case from Morgagni; and this part of the subject concludes with some practical remarks. These are very few. The principal caution proposed is, lest the "disease should terminate in chronic inflammation, which rarely fails to carry off the patient in a few days." If so, we should be disposed to call such an inflammation acute. We have, however, no objection to make to the proposed practice; which does not differ materially from what is usually attempted under such symptoms.

A chapter follows, on the chronic inflammation of the heart; in which we are informed, that it often resembles carditis, though essentially different from it. For our parts, who are not equal to all these subtleties, we should be disposed to consider the diseases as only differing in degree; and when one of them is running through its stages in a few days, we should be unwilling to tell our patient that he had a chronic disease, or to treat it as such. Adhesion to the pericardium, if firm, we should consider as the effect of strong action or high inflammation. If after this, or any other such effect, the actions of the heart should become irregular, the disease might be truly called chronic; but this would be the effect of the original acute disease. Such a disease shows a constitutional disposition to inflammation, and perhaps to inflammation in that organ. Nothing, therefore, can be more probable, than that acute inflammation, suddenly supervening on such an imperfect state of parts, should, in a short time, prove fatal.

The succeeding section, on the effects produced by the diminution of the heart, is extremely imperfect, from the want of distinguishing, with any accuracy, the probability of original formation and the effect of disease. Under any difficulty of action to a muscle, the consequence must be, either an increase of muscular fibres and strength, or a decrease and weakness. If the organ permanently attempts to overcome the difficulty, the consequence will be an increase of its muscular fibres and powers, as we observed before of the bladder, in obstructions to the passage of the urine. But if the difficulty is attended with pain, especially at every over exertion, as is most remarkably the case in diseases of the heart, the attempt will then be, to avoid such over exertion as much as possible; and the patient will shun all violent exercise or passions, which would increase the action of the heart. Hence the volume and force of its muscular fibres are diminished. Such appears to have been the case with Mr. Hunter. "On the under surfaces of the left auricle and ventricle, says Mr. Home, were two spaces of an opake white appearance, covered with coagulating lymph, the result of former inflammation."—"The heart itself was small, appearing as if it had shrunk. The muscular structure was paler and looser than the other muscles of the body."—The pains taken by that philosopher to prevent the too great action of the heart, are well known; and to this we may fairly impute its diminished size and power.

The chapter on the consequences resulting from change of structure of the substance of the heart, contains some interesting remarks, which serve to confirm what Mr. Hunter justly observes, that a heart cannot be essentially necessary for circulating the blood.

The observations on disease of the coronary arteries, and on syncope anginosa, follow. By the title of this chapter, the reader will perceive that the author imputes syncope anginosa, or angina pectoris, to diseases of the coronary arteries. By their ossification, he conceives that the heart, deprived of its due portion of blood on any occasion for increased action, not only is unequal to that increase, but, in the attempt, ceases to act altogether. To this want of nutriment also, is imputed the diminished size of the heart.

Having, in justice to the author, explained this theory, we shall leave the decision to the reader. The practical remarks that follow, seem, in many respects, judicious; but we cannot help wondering that Dr. Perry's admitting the occasional use of stimuli, should be considered as a concession to the doctrines of others. In some cases of angina pectoris, it is well known that immediate application to brandy has always restored the patient; and that some have been supposed to die under the syncope, from having failed to provide themselves with that necessary recruit. But these paroxysms are not to be confounded with those more acute and painful painful ones, which are probably true inflammation, and lay the foundation of the chronic misery.

A chapter follows on polypi, well worth perusing, as it marks the difference pretty accurately between those which are formed after death, or in articulo mortis; and those which, arising from high inflammation, become organized. But still we are at a loss to conceive how our author can explain many of the symptoms, and of the appearances, without admitting high inflammation as the cause; and, consequently, why under any such circumstances, he should object to venesection. Withont entering into this controversy, we shall transcribe the following case, as a fair illustration of the organization of these polypi.

"To these proofs of the vitality of some polypi, I may add another, deduced from the diseased state in which we found the polypus itself, in a person who had evidently died from angina pectoris. In this subject, the nutrient arteries of the heart were extensively ossified; the aortic valves had fungi attached to their floating margins, and one of the chordæ tendineæ was implanted in such a way into one of the semi-lunar valves, as to destroy in a great measure its valvular action. In the left ventricle, a polypus concretion which measured more than one inch in length, was found attached to the septum of the heart. That side of the polypus next to the septum adhered so firmly to it, that the membrane lining the ventricle tore, before the polypus could be separated from the heart. Where the two were in contact, the septum was painted with small red vessels, and was besides rough. This fact of the firm union of the polypus with the heart, is a sufficient proof of the vitality of the latter; but this is established beyond a doubt, by an abscess being found in the centre of the polypus itself. This abscess, when opened, discharged above a tea-spoonful of perfectly formed purulent matter.

"In this dissection, I would particularly call your attention to the existence of the polypus, and the diseased nutrient arteries. Can we have a more unexceptionable proof than this dissection affords, of the causes which predispose to the formation of polypi; and is it possible to procure a more satisfactory argument in favour of the vitality of some polypi, than the one furnished by this subject? The ossification of the coronary arteries, and their otherwise diseased state, clearly shew, that the action of this heart must have been languid; and the presence of a fully organized polypus, corroborates a position formerly laid down: it tends to establish the point, that impaired action of the heart predisposes to the formation of polypi. The existence of the abscess in the centre of the polypus, surely shews that the concretion had been endowed with vitality: it also, I think, proves that the polypus must have existed a considerable time before death.

"The polypi we have been just describing, are, without doubt, merely formed from the blood changed by the action of the vessels of the heart, and supported by twigs from the coronary vessels;

 but but there is another species of concretion occasionally met with in the heart, where the cavity in which it is contained, is much more extensively affected. In this disease, the parietes of the heart are generally thickened, in spots ossified, and the inner surface of the cavity is rugged, and lined with the same kind of substance which invests the inside of aneurismal tumors. This species of concretion has very much the look of being a fungus from the heart itself, and is altogether unlike the other polypi, which seem productions in the first instance from the blood contained in the cavities of the heart. We have in our possession a heart, where the concretions is as large as a pullet's egg, and where it is attached by a broad base to the side of the left auricle, which, over its whole extent, is thickened and diseased; and in this instance, by forcing air into the coronary vein, we could inflate some few vessels in the new formed substance, in which there were likewise some specks of bone. In this person, the mitral valve was so malformed, that the presence of the polypus in the auricle, was a matter of complete indifference. Indeed, as has already been noticed, we have great reason to doubt, whether a case of idiopathic polypus of the heart has ever occurred. If blended with other diseases, its presence can rarely before death be ascertained; and if discovered, it would not alter the plan of treatment."

On the effects resulting from ossification of the valves, we meet but little novelty; nor, indeed, is it reasonable to expect it.

The chapter on aneurism is principally a compilation, and chiefly from the ingenious and indefatigable Scarpa. We should remark, that in this and some other chapters, our author has much credit in marking the anomalous or sympathetic symptoms.

The remarks on preternatural pulsation in the epigastrium form a good compilation, but might have been extended further. Whytt, in his account of nervous diseases, has some observations which are well worth notice; particularly as they were written before several pathological facts were known, which are now well ascertained.

After all that is said, this subject is not yet cleared of obscurity; in other words, there are cases in which a collection of feculent matter, to a very great quantity, has been attended with no pulsation; and others, in which this pulsation seems, if not to arise from a subsultus of the muscles, to be at least ascribable to no ascertained cause.

The last chapter contains, "remarks on the unusual origin and course of some of the large and important arteries of the human body." Where the aorta deviates so far from its natural course as to receive venous blood, the consequences are too well known. In other instances, where changes in the disposition of the arteries take place at parts within the sphere of chirurgical operations, every caution is absolutely necessary; and the surgeon should always be aware of the possibility of such an event. But we cannot help doubting how far dysphagia should follow from any irregularity gularity in the ramifications of the aorta, how great so ever they may be.

Such is the nature of this work, which, though far from being as complete as we expected from an anatomical teacher, is certainly not without its merit. In a few years we suspect the author will alter his opinions on some subjects, and become more decided in others. In the meanwhile, we are pleased that the work has appeared; and hope it will lead to a more minute examination of every malformation, or change of structure, which may occur in these important organs.

---

### Edinburgh Journal, No. XX.

( Concluded from our last, pp. 427—433. )

Article 5.—*Case of preternatural Enlargement of the Heart, with diseased Pericardium.* By Charles Aldis, Surgeon.

This case was attended with ossified valves, and a beginning ossification of the aorta. The pericardium was every where adherent, and a portion of it is said to have been in a highly morbid state, but its disease is not described.

Article 6.—*Observations on Contagion. Communicated to Dr. Chisholm, from Dr. David Hossack.*

As far as we can understand Dr. Hossack, he wishes to show that the distinction between infection and contagion may be better reconciled to the mind, by dividing contagions into different classes. The first are those which are communicable by contact only, viz. The Itch; Syphilis; the Sibbens of Scotland; the Laanda of Africa; Framboesia or Yaws; Elephantiasis or Leprosy; Hydrophobia, and the vaccine virus." The second are communicable by contact and by the atmosphere, as Small-pox; Measles; Chicken-pox; Influenza; Hooping-Cough; Scarlet Fever, and Cynanche Maligna." The third by the atmosphere only, as "Plague; Yellow Fever; Typhus, *Jail, Ship, Hospital or Lake Fever*, and Dysentery.

We would first remark, that we have never heard of influenza being communicated by contact. If contagious at all, it seems to us reduceable to the same class as yellow fever and plague, that is, spreading only under a certain constitution of the atmosphere. All three attack suddenly a great number of subjects, after which, they as suddenly disappear, not like the small pox, because there are no more subjects to be attacked by them, but because of the change in the atmosphere. This is remarkable in influenza, which rarely continues more than a month or six weeks in any one place. The yellow fever usually subsides suddenly on the access of heavy rains; the plague in Egypt always ceases with the summer solstice, and in Europe with the winter frost.

Contagion, which is derived from *contangere*, implies contact, whether ther of the person himself, or of the air which has touched him, is of little consequence, as to the general term, though highly proper to be marked in distinguishing the different laws of each. Infection, on the contrary, from *inficere*, is of a more general import, implying only that certain properties are imparted, by which the air or any other substance is altered. If this alteration is such as to enable the *infected* air to produce disease, it may then very properly be called *infectious*. But under infection the air does not necessarily convey the same disease as the patient suffers, and contact of the person where the air is not infected is no way injurious. A number of persons confined with any disease will so far injure or infect the air, that it shall prove infectious or induce fever in those who breathe it, but persons thus infected will not prove injurious to others.

Hence, both the etymology and analogy fails, because the air that touches a man under this disease, and is breathed by another, does not induce the disease in the latter. The magistrates who have been infected by gaol fevers from the infectious air of a prison, never prove injurious to others. But the subject under scarlet fever or small-pox, however well ventilated the place may be, proves contagious to all who are susceptible of the disease. In the yellow fever, it is found, that those who remove from infectious towns are not injurious to others.

We would not, however, quarrel about terms, for if the classes of diseases are accurately distinguished, it is of little consequence by what name. But the practical inference, which is the most important, seems too little attended to by our author.

"This doctrine, too, I believe, says he, will better account for the apparently contradictory facts, which have been urged by the advocates of the two opposing opinions, than any system that has been adopted.

"It will also lead to a system of police regulations, which will best insure us against the ravages of yellow fever when introduced; at the same time, that it will teach us carefully to guard against the introduction of it from abroad."

Now this last paragraph seems to us likely to perpetuate all the errors which have so long proved the bane of North America. If, as is universally admitted, the yellow fever can only exist at certain seasons, so that quarantines are only enacted at those seasons, and if no quarantines have been sufficient to protect the ports from the disease at certain times; if at these seasons, refugees from the towns have never conveyed the disease into the country, it seems doubtful whether quarantines are of any use: in either case, the first object should be to ascertain whether the disease is imported at all; and if not, to make such provision for draining their towns, as may preserve them from this calamity.

Article 7.—*Cases of Enlargement of the Knee Joint, cured by the Application of Corrosive Sublimate.*—By William Robertson, one of the Surgeons of the Kelso Dispensary.

This paper contains some good practical remarks, which convince us, that the author will not be offended if we attempt to correct some erroneous opinions, the impressions excited by which, may have an improper influence on the reader. In the exordium to the cases, we have the following among other remarks on the disease.

"The ligaments, cartilages, and bones, being of an irritable nature, are susceptible of inflammation; and if this be not subdued by the means employed for resolution, it passes into the suppurative state, which destroys the connecting medium of the bones, and renders the waste of the parts occasioned by suppuration irreparable. Under such circumstances, if the ends of the bones do not unite by a process of nature which has got the name of *anchylosis*, which however seldom if ever happens unless to young people, the patient becomes hectic, in consequence of the absorption of matter from the joint, and, if he does not submit to the amputation of the limb, he lingers out a life of misery, till death puts an end to his sufferings."

We had conceived that this opinion concerning the cause of hectic fever in incurable diseases, had been so long banished from the London school, as by this time to become obsolete in most parts of the Empire. We only wish to remind the author, that when suppuration has taken place in such a cavity, the most desirable event is, that the *whole of the matter* should be absorbed without any breach of the external skin, and that when such an event takes place, we have no hectic fever. In more unhappy cases, the hectic rarely takes place till the skin is broken, consequently till the absorption is probably less; but repeated suppurations over a large surface, in order to heal by the common process, produces a perpetual recurrence of symptomatic fever, till it becomes a true hectic. If this were not the case, it would be inexcusable in surgeons ever to permit matter to remain without free openings, and the application of absorbent substances all over the surface. But the danger of opening extensive and deep seated abceesses was so long known, that surgeons were taught by the consequences to avoid it as much as possible. In the psoas abscess the patient's case was considered as almost hopeless, when the matter proceeded to the surface, and an external opening was made by ulceration or art. The danger in these cases was in proportion to the surface of the abscess, which till opened, would gradually increase. Mr. Abernethy, therefore, made an important practical improvement in this part of surgery, by opening such abscesses at an early period, and suffering the orifice to heal. In these cases it sometimes happens that the stimulus given to the surface of the abscess is sufficient to induce a healing process; that is, a fresh suppuration takes place over the whole; the the matter secreted is absorbed; the sides of the abscess unite by granulation, and the patient is cured. During this whole process, nothing is so little apprehended as the absorption of matter.

The next error which we would point out to our author, is his manner of confounding highly irritating substances with *caustics.* This is particularly important, because a part of his success seems to have arisen from the very high inflammation he excited externally, where it was manageable, and which probably relieved the parts within. A caustic is literally a substance which burns the part, and by that means produces its destruction. We are, however, in the habit of using the term for such substances, as by entering chemically into combination with the animal matter to which it is applied, produces such a change as to render it unfit for its former purposes; in consequence of which, it is separated by a future process of suppuration. As these substances act alike on all the soft parts of animals, whether dead or living, they may be applied over the sound cuticle. Corrosive sublimate and arsenic have none of these properties: when applied to parts having sensibility, they excite so high an inflammation as the parts cannot support, and death is the consequence; that is, mortification follows, and the part must afterwards be separated in the same manner as after the application of chemical caustics. This last circumstance may seem to render the distinction of less importance, but this is far from the case.

The chemical caustics, we remarked, may be applied efficaciously to the sound cuticle, because their action does not depend on the sensibility of the part. But the mineral poisons, if we may so call them, will produce no caustic effect, excepting the skin is broken or previously scarified, so that they may come into contact with parts endued with sensibility. The consequence is very obvious. If we wish merely to make on opening into an abscess, or any other cavity, and that our opening shall be such as to prevent an immediate re-union, the chemical caustic is the most convenient. To this we must impute the whole success of Mr. Else's method of curing hydrocele. If the mineral caustic had been used, and the skin divided previous to that operation, the inflammation excited would have been so great, that extended over such a surface as the enlarged and highly sensitive tunica vaginalis, would probably have proved fatal.

In the cases related by Mr. Robertson, the inflammation excited by the chemical caustics might have been unequal to the purpose, whilst the high inflammation which was excited by corrosive sublimate was attended with so many advantages.

We shall conclude these remarks by adding, that we think the faculty obliged to the author of this paper for the introduction of this substance as a topical application, and we are particularly pleased with his candour and habits of observation, which are so conspicuous, in his acknowledging the sources from which he derived so useful a piece of information.

ARTICLE

ARTICLE 8.—*Observations on* Mr. BARLOW's *Theory on the Origin of Urinary Calculi.* By WILLIAM GOODLAD, Member of the Royal College of Surgeons, London.

THEORIES, and observations on them, appear to us, rather as lessons for themes than really useful for practical knowledge. We do not, however, entirely condemn them. Reasoning cannot be long supported without producing some facts; the authenticity and accuracy of which, will be established by the keenness of the opponents.

*The Inquirer, No. XII.*

Are the remote Causes of Stone and Gravel yet discovered?

THIS is a judicious and well written Essay. The difficulty the author finds in drawing any conclusions, may serve as a further apology for the little notice we took of the preceding paper, and as a lesson to the antagonists.

---

*Practical Observations on Strictures of the Urethra, with Cases illustrative of the comparative merit of the caustic and common bougie, also Remarks on Fistula in Ano, and an improved method of treating Tinea Capitis, with annexed Cases*; by THOMAS LUXMOORE, Surgeon Extraordinary to the Prince of Wales; Surgeon to the Eastern Dispensary, &c. &c. 8vo, pp. 215, London 1809.

IN an introductory chapter, Mr. Luxmoore, after remarking the imperfect knowledge in stricture before Mr. Hunter's time, observes, that "Mr. Home has written well on this subject," but seems to apprehend, that "his better judgment may have been warped by his attachment to Mr. Hunter, in favour of a practice, on which he has made great improvement, though it ought not to be adopted without much discrimination." We confess ourselves somewhat at a loss to comprehend clearly the meaning of this passage. If Mr. Home has improved Mr. Hunter's practice, it must be by a much more free and general use of the caustic than Mr. Hunter proposed, which is very different in our opinion from any bias in favour of his master. In saying this, we pretend not to determine the propriety of either practice, whilst we may truly say, *adhuc sub judice lis est.*

"Although, continues Mr. Luxmoore, it be universally admitted that bougies are indipensable for the cure of strictures, still, the application of these instruments does not seem to have been clearly pointed out, nor their principles of action sufficiently understood. The following observations are therefore offered to the public, as the result of much experience.

"The advantages afforded to the author, in the public institution committed to his charge, have furnished ample opportunities of treating the complaint which is the subject of the present publication, in every form and variety, and according to the various modes recommended; nor have his opportunities of observation in private practice been confined: the chief object of these remarks is to limit the use of caustic bougies.

"Mr.

"Mr. Home has, it is true, in his judicious publication, pointed out the cases to which caustic chiefly applies; but in doing so, it was evidently not his intention to recommend its application so indiscriminately as it is commonly employed; for every surgeon of experience knows, that not one case in ten requires its application; and that the generality of practitioners, not perhaps reflecting sufficiently on the rules which Mr. Home has laid down, are apt to employ a remedy which, in unskilful hands, is attended with the most dangerous consequences."

That the principles of action of bougies was not sufficiently understood, we are ready to admit; but after Mr. Hunter's explanation, and Mr. Home's further improvement, it did not increase our expectations of the merit of the work before us, to find the author announcing the advantages his establishment afforded him.

The first chapter on the stricture of the urethra cannot be expected to contain much novelty.

The second chapter is on the origin and formation of stricture. Among the first of these, inflammation, and the consequent deposition of coagulable lymph which becomes organized, is mentioned. This we should suspect would occasion caruncles, which it is now admitted are very rare. The author indeed informs us, that the deposition is in the cellular membrane, and that circular bands are the consequence. This may be the case, but it is difficult to conceive how so regular a process should follow common inflammation, and the effusion of coagulable lymph. An opinion is expressed too, that Mr. Hunter must be mistaken, in doubting whether stimulating injections ever produce the stricture. The next cause remarked by our author is that mode of protracting the venereal act, mentioned by Mr. Home. After this follow some remarks on the spasmodic stricture, and its connection with the permanent.

The third chapter, on the symptoms of stricture, cannot be expected to contain much novelty.

A chapter follows on diseases resembling stricture. These are the diseased prostate gland, and the irritable bladder, the various forms of which are related with attention, and some good practical inferences follow.

The consequences of stricture form the fifth chapter; those mentioned by the author are, the thickening of the bladder; the gleet; and fistulæ in perinæo.

Mr. Luxmoore next enters into a short history of the caustic bougie, tracing it from Ambrose Paré, to Mr. Hunter, Messrs. Home, Whately, and Andrews, and shewing very briefly the inconveniences which sometimes occur in its use, and also, that it cannot be considered as a permanent cure, with any greater certainty than the common bougie. Some remarks on the common bougie follow, their various improvements, and the best mode of applying them. The author prefers the plaister bougie. After a few other remarks on some diseases of the prostate gland, he proceeds to illustrate the preceding observations by his own practice, producing cing cases of most of the diseases above enumerated. We extract the following passage, as containing two remarks we think it right to produce to our readers, but shall offer no commentary of our own.

"The above case is chiefly to be remarked, inasmuch as the cause of stricture was evidently the use of astringent injections. It has been strongly asserted, by Mr. Hunter, that this could never occasion the disease; but so many proofs of this kind come before the surgeon, that I believe his opinion is more ingenious than well founded. In this case, I first followed the common mode of using a small bougie, but experienced no success from this effort; on the contrary, I found the application of a middle sized one, to be the only means of gaining any ground. In my attempts here, I deviated a little from my usual plan, and employed more force in pressing against the stricture, than I have recommended. The consequence of this was, that I gained ground, and passed the bougie suddenly for an inch through the stricture, but at the same time it produced such a degree of irritation, that the patient became faint. Soreness prevented my proceeding; and, on making an attempt the following day, I could not pass the stricture. These trials were repeated on the subsequent days, and it was only by regular perseverance, in pressing upon the stricture for a certain period, that I at length succeeded in procuring a passage into the bladder. This clearly shews, that the mild method of procedure is both the safest and most successful; and that a gradual and continued pressure will overcome difficulties apparently insurmountable; for in this case it required eight weeks to procure a free passage."

If we have had occasion to remark, that little novelty or information can be gained by the perusal of this part of the work, it is but justice to add, that the succeeding chapters on Tinea Capitis have considerable merit. If we have to complain of Mr. Luxmoore, as of most other outhors, that the disease of which he treats is ill defined, it must be admitted, that he has made those divisions of complaints in the scalp, which will very much assist the reader, in discriminating the various symptoms, and applying remedies respectively to each.

"I consider," says he, "the disease divisible into two species, viz. the dry and the moist. The dry is the more obstinate, and is vulgarly called *ring worm*, in which the hair drops off in patches. It generally resists every means employed for its cure, but in some cases a temporary relief seems to be obtained. Three cases lately occurred, sent me by Mr. Astley Cooper, which yielded to the treatment employed, and were ultimately completely cured.

"The moist form occurs more frequently, and generally engages most attention. In this species, the Cutis Vera is the seat of the disease; it seems to have its origin in the bulbs of the hairs, and from these, extends to the circumjacent cutis. Some cases which occurred occurred in negro children, gave me an opportunity of remarking that the rete mucosum, was not reproduced when the sores healed.

"The existence of animalculæ, as supposed by professor Redi and others, has not yet been proved, and I am inclined to consider it an erroneous idea. The causes of this disease may be considered of two kinds; those which act generally, and seem to predispose to the disease, and those which act topically, and excite the complaint.

"Of the first kind, seem to be scrophulous diathesis, poorness of living, and want of cleanliness.

"Of the latter kind, filth, and the matter itself which is produced from cases of Tinea. There is no doubt but the matter is capable of exciting the disease in others, and I believe that the complaint is most commonly occasioned by contagion. Whether it ever originates merely from neglect and want, I have not been able to determine; since, if the subject be of the higher classes of society, it may generally be traced to a source of contagion, and if of the lower orders, both causes seem to operate. There can, however, be no doubt but that negligence, and inattention to the means of cleanliness, certainly aggravate the complaint. Such being the case, it is absolutely necessary in the treatment to attend to cleanliness; but I do not conceive that it should form the chief indication in the plan of cure, as has been often maintained.

"The idea of this disease, which I wish to enforce, is, that it consists of a chronic inflammation, productive of a secretion of matter peculiar in its nature, and capable of propagating the complaint if applied to the scalp of a healthy subject, as much so as that of syphilis and other specific diseases. If applied to other parts of the body, it will also produce a similar disease, allowing for the difference in the structure of the skin of those parts, and which is generally known under the name of ring-worm. The lymphatic glands, in the vicinity of the affected part of the scalp, become swollen and painful, probably from the absorption of this matter."

The author then proceeds to mark the constitutional symptoms which attend this local disease, and also the remedies by which they are to be relieved; several striking cases are annexed, and, on the whole, we cannot but express our satisfaction at this part of the work.

In his remarks on Fistula in Ano, we confess ourselves unable to follow the author through every part. It may, perhaps, be judicious to remind practitioners of the necessity of attending to the constitution under all local diseases; but where the general health is good, we find it difficult to trace a local disease to the imperfect action of any distant organ.

"The first and most frequent cause of this complaint, says our author, is a scrophulous habit, and is often accompanied with a diseased diseased state of the lungs. In such a constitution, any slight injury in the vicinity of the rectum, occasioning inflammation, will produce this affection, and whatever local means may be employed, will be totally ineffectual, unless the primary fault of the constitution be repaired. The determination to the skin in such persons is less than natural; the consequence of which must be, that the vessels of the intestines, whether arteries or veins, will become more loaded with blood. The blood-vessels themselves are also of a more relaxed structure; hence they dilate considerably, and thus the circulation is rendered extremely languid, particularly in veins, where the blood acts against its own gravity. The consequence of this is, that the blood from the rectum, and its neighbourhood, is impeded in its return to the heart. The congestion thus produced, will occasion the surrounding cellular membrane to become laden with fluid, produced from the exhalent terminations of the arteries; while the veins, from their increased size, occasioning a mechanical pressure and irritation, will, as they enlarge, break down the cells of this membrane, and excite in it an erisypelatous inflammation; the matter then frequently points externally in the form of a tumour, or it is discharged with the stools. On introducing a probe, the rectum is found denuded, or the sinus penetrates either into the nates or perinæum.

"Another cause of this malady may be traced to an imperfect cure of syphilis, nor does it unfrequently proceed from the injudicious use of mercury in a scrophulous constitution. It is therefore of much importance to ascertain which has given rise to the disease; and to determine this, recourse must be had to the history of the case. When the cause is venereal, mercury must necessarily be employed, or the operation, conducted in the most skilful manner, will certainly disappoint the wishes of the surgeon. If, on the contrary, too much mercury has been given, the constitution will be so debilitated, as to render it necessary that tonic remedies, nutritious diet, and good air, should be recommended, previous to the performance of the operation."

The third cause mentioned by Mr. Luxmoore, appears to be most general, viz. erisypelatous inflammation. When this disease attacks the parts in the neighbourhood of the rectum, the consequence must be those sinusses which form the true character of the disease. Venereal sores never assume that form. If mercury has exasperated them, they have usually been fistulous before that remedy was used. That the constitution should be attended to under all local diseases, cannot be questioned; but, when that has appeared sound, we have never found reason to repent directing our whole attention to the local disease.

We here take our leave of Mr. Luxmoore, not without recommending some parts of his book to the attention of our readers; may we add, if that which relates to strictures had been omitted, we do not think the reputation of the author, or the value of the book, would have suffered.

